# Prevalence and risk factors of suicide and their correlation with depression and anxiety among medical students: a Saudi multicenter study

**DOI:** 10.3389/fpsyt.2026.1879787

**Published:** 2026-08-07

**Authors:** Ayedh H. Alghamdi, Ahmad H. Almadani, Mohammed A. Aljaffer, Saleh A. AlGhamdi, Abdulmajeed A. Aldrees, Mohammed A. Almansour, Nihal A. Almuraikhi, Hamzah A. Al Fiaar, Elzahraa O. Ibrahim, Amal M. Alshadwi, Hussam S. Ashetwi, Fatima A. Alhaidar

**Affiliations:** 1 Department of Psychiatry, College of Medicine, King Saud University, Riyadh, Saudi Arabia; 2 Psychiatry Department, College of Medicine, Imam Mohammad Ibn Saud Islamic University (IMSIU), Riyadh, Saudi Arabia; 3 Departments of Physiology, College of Medicine, King Saud University, Riyadh, Saudi Arabia; 4 College of Medicine, Dar Al Uloom University, Riyadh, Saudi Arabia; 5 Medical Education Department, College of Medicine, King Saud University, Riyadh, Saudi Arabia; 6 Stem Cell Unit, Department of Anatomy, College of Medicine, King Saud University, Riyadh, Saudi Arabia; 7 Eradah Complex for Mental Health, Third Health Cluster, Riyadh, Saudi Arabia; 8 Department of Psychiatry, King Saud University Medical City, King Saud University, Riyadh, Saudi Arabia; 9 Eradah Hospital for Mental Health, Ministry of Health, Jazan, Saudi Arabia

**Keywords:** anxiety, depression, medical training, mental health, Saudi Arabia, students, suicidality

## Abstract

**Introduction:**

Medical training is a major source of stress for medical students, potentially affecting their mental well-being, and subsequently increasing their risk of mental disorders and suicide. This study examined the prevalence and risk factors of suicidality among medical students at four universities in Riyadh, Saudi Arabia.

**Methods:**

A cross-sectional study was conducted. The study tool consisted of a questionnaire developed by the research team along with the Patient Health Questionnaire-9 (PHQ-9), General Anxiety Disorder-7 (GAD-7), Columbia-Suicide Severity Rating Scale (C-SSRS), and Brief Resilient Coping Scale (BRCS).

**Results:**

The study included 450 medical students (251 females and 199 males). According to the C-SSRS, 28% of the participants reported death wishes, 8.2% reported suicidal ideation during the past month, and 10% were classified as having a moderate-to-high suicidality risk. Depressive symptoms were present in 38.7% of the participants, including 16.5% with moderately severe to severe depression; 61.8% reported anxiety, with 32.2% experiencing moderate to severe levels. Multivariable logistic regression identified severe depression (aOR = 14.54, 95% CI: 3.09–68.55), a history of abuse of any form (aOR = 4.22, 95% CI: 2.02–8.84), and mild anxiety (aOR = 3.29, 95% CI: 1.08–10.01) as independent predictors of moderate/high suicidality risk.

**Conclusion:**

Our findings demonstrate that suicidality is a concern among medical students and is associated with depression, anxiety, and other factors. These findings highlight the need to promote prevention and early detection to facilitate access to timely management, including mental health services.

## 1 Introduction

Suicide is defined as death caused by self-directed injurious behavior with the intent to die as a consequence of that behavior (1–3). Suicide is a major global public health concern. According to the World Health Organization (WHO), more than 700, 000 individuals die from suicide each year worldwide. Similarly, the Centers for Disease Control and Prevention reported more than 49, 000 suicide deaths in the United States alone in 2023 (1, 2). Suicide is currently the fourth leading cause of death among young men and women, aged 15–29 years (1).

Suicide is a major global public health concern, with nearly 77% of suicide deaths occurring in low- and middle-income countries where mental health resources are often limited (1). The Kingdom of Saudi Arabia (KSA), despite being a high-income country, also reports suicide cases at an estimated national rate of approximately 1.1 per 100, 000 people annually (4). Research on suicidal behavior in Muslim-majority societies remains relatively scarce. However, available evidence suggests that suicidal ideation and attempts occur within these populations, though they may be underreported due to cultural and social factors (5). For instance, data reported by the Saudi Ministry of Health (MOH) documented 45 suicide deaths within MOH facilities between 2011 and 2020 (3).

Suicidal behaviors are multifactorial and arise from complex interactions among social, psychological, cultural, and clinical determinants (6–10). Well-established risk factors include depression, anxiety, burnout, sleep disturbances, prior psychiatric conditions, and family history of mental illness or suicide (7, 8, 10). Additional contributors, such as limited social support, social isolation, interpersonal conflict, substance misuse, academic or financial stress, and chronic pain, may further increase vulnerability (6–10). Importantly, suicidal behavior is rarely attributable to a single precipitating event. Rather, it typically reflects the cumulative effect of multiple interacting stressors that together increase the likelihood of suicidal behavior (6).

Certain populations are more vulnerable to suicide than others. Suicide-related phenomena (including suicidal ideation, planning, and attempts), psychological distress, and depression are reported at notably higher rates among college students, especially those enrolled in health-related programs, such as medicine, nursing, and dentistry (7, 8, 11–15). ​ Evidence consistently indicates that elevated levels of stress, anxiety, and depression among healthcare students, which may increase their susceptibility to suicidal ideation and attempts (16).​ Compared to age-matched peers in other academic disciplines, students in health-related disciplines consistently exhibit higher levels of psychological distress (8–14). Medical students are among the most vulnerable populations because of the demanding medical curricula (7, 8, 11, 13, 14). Heightened vulnerability is often attributed to the substantial academic workload, competitive learning environment, frequent high-stakes examinations, long study hours, and emotional demands associated with clinical training and patient care. Clearly, mental health during medical education is an important area of concern (17–20). Consequently, higher levels of stress, anxiety, burnout, and depressive symptoms have been consistently reported among medical students than their peers in other academic disciplines (7, 13, 14, 20, 21). Additionally, medical students exhibit higher rates of suicidal ideation than the general population, with prevalence estimates ranging from 1.8% to 53.6% across different settings (12, 13, 17, 20–24). Some studies have further indicated that female medical students report higher rates of suicidal ideation and attempts than male medical students (8, 11, 13, 20).

In Saudi Arabia, similar patterns have been observed, with studies reporting substantial levels of psychological distress among medical students, including prevalences of approximately 57% for stress and 16% for depression (19, 21, 25). Collectively, these findings highlight that the mental health of medical students and future physicians is an increasingly pressing concern (25).

In Saudi Arabia, research examining suicidal behaviors among medical students remains relatively limited. The reported prevalence of suicidal ideation varies considerably across studies (4, 26). Furthermore, few studies have explored suicide risk within the broader context of the medical education environment in Saudi medical schools. Given the unique academic pressures and demanding learning environments associated with medical schools, further investigation is warranted. This study aimed to estimate the prevalence of suicidal behaviors among undergraduate medical students and the potential risk factors, including symptoms of depression and anxiety, in different medical schools in Riyadh, Saudi Arabia.

## 2 Materials and methods

### 2.1 Study design, setting, and participants

This multicenter cross-sectional study was conducted among undergraduate medical students enrolled at four universities in Riyadh: King Saud University (KSU), Imam Mohammad Ibn Saud Islamic University (IMSIU), Vision College, and Dar Al Uloom University (DAU). Data were collected between November and December 2025. The inclusion criteria were all male and female undergraduate medical students across all medical college years (preclinical and clinical phases) enrolled at participating universities during the 2025–2026 academic year. Students were excluded if they were in their internship training year, enrolled in non-medical colleges, or studying at institutions other than the four participating universities.

For each of the four targeted universities, the corresponding Academic Affairs Office provided an Excel spreadsheet listing all eligible students. Subsequently, the research team revised the Excel sheet received from each university, subdividing the data into two spreadsheets: one for male students and one for female students. The research team also rearranged the lists according to academic year. After the year-based rearrangement, random numbers were generated in Excel using the RANDBETWEEN(1, N) function, and the lists were sorted accordingly. Subsequently, based on the estimated required sample size from each university, the research team recruited participants listed in the randomized final spreadsheets. The required sample size from each university was calculated based on the size of the targeted population of each university, as provided initially by the corresponding Academic Affairs Office.

**Figure 1:**
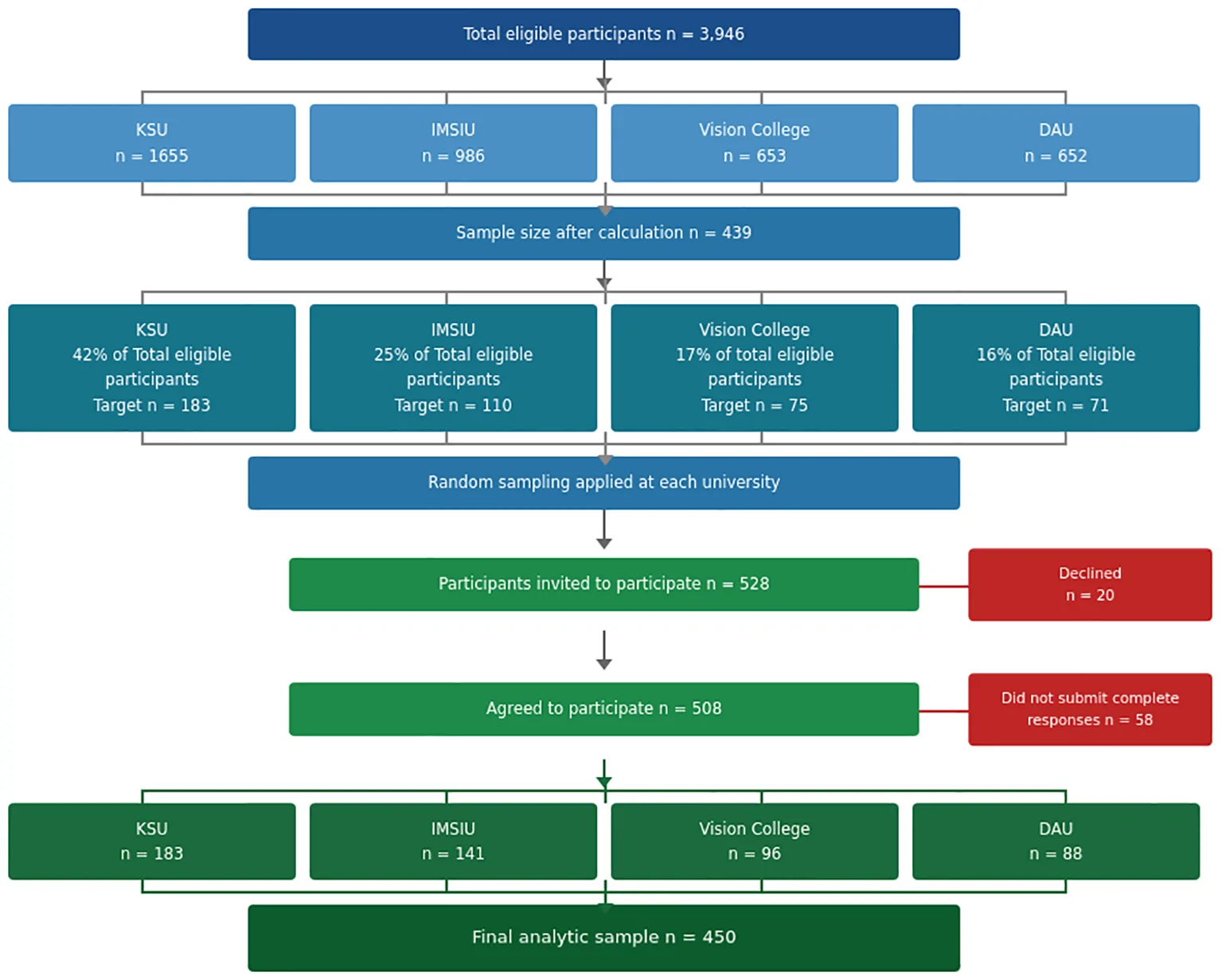
Recruitment and sampling process flow diagram.

To ensure representativeness across institutions, the research team calculated the total number of eligible students across the target universities as 3946. Based on this figure, we calculated the overall required sample size as 351 students and added 25% to account for incomplete responses, adjusting the targeted sample size to n = 439. Based on the figures from each university’s Academic Affairs Office, we calculated the required sample size for each university as follows: KSU (42%, target n = 183), IMSIU (25%, target n = 110), Vision College (17%, target n = 75), and DAU (16%, target n = 71). **Figure 1** illustrates the recruitment and sampling process. The sample size was calculated using the Raosoft online sample size calculator (www.raosoft.com), assuming a 95% confidence level, 5% margin of error, and expected prevalence of 50%.

Participants from each university were determined from the final randomized spreadsheet, noting that the next person on the list would be contacted if the potential participant did not respond to our repeated phone calls or refused to participate during the actual call. During each phone call, the research team explained the study to the potential participant and invited them to participate. Those who agreed to participate in the research were sent the study tool’s link via WhatsApp or an official email, whichever they preferred. On the initial survey page, official consent was obtained from each participant by clicking the “agree to participate” button after reading the informed consent. Participation was voluntary and anonymous.

### 2.2 Study instruments

Data were collected using an anonymous electronic self-administered questionnaire developed on the SurveyMonkey platform. The survey consisted of two components. The first section was developed by the research team to obtain sociodemographic and background information. Sociodemographic variables included demographic characteristics (age, sex, marital status, living arrangement, and family income), academic characteristics (type of university and academic year), health-related factors (level of physical activity and history of chronic medical illness), mental health history (previous psychiatric diagnoses, use of psychiatric medications, or psychotherapy), and psychosocial factors (family history of psychiatric disorders or suicidality, exposure to recent life stressors, and perceived social support). The second section included a set of validated psychometric instruments used to assess depression, anxiety, suicidality, and psychological resilience.

Depressive symptoms were assessed using the English version of the Patient Health Questionnaire-9 (PHQ-9), a validated and widely used screening tool consisting of nine items measuring depressive symptoms during the preceding two weeks. Each item was scored on a 4-point Likert scale (0–3), yielding a total score ranging from 0 to 27. Depression severity was categorized as minimal (0–4), mild (5–9), moderate (10–14), moderately severe (15–19), or severe (20–27) (27–29).

Anxiety symptoms were evaluated using the English version of the Generalized Anxiety Disorder-7 (GAD-7) scale. The GAD-7 is a self-administered seven-item instrument assessing anxiety symptoms over the previous two weeks. Each item is scored from 0 to 3, yielding a total score ranging from 0 to 21. The severity of anxiety was classified as mild (5–9), moderate (10–14), or severe (15–21) (29, 30).

Suicidal ideation and behavior were evaluated using the English version of the Columbia Suicide Severity Rating Scale (C-SSRS), a validated and widely used tool in clinical and research settings. The C-SSRS assesses a spectrum of suicidal thoughts and behaviors, including passive death wishes, active suicidal ideation, suicidal planning, and suicidal behaviors. Based on the predefined severity classifications of this instrument (C-SSRS), participants were categorized into no/low suicidality risk, moderate risk, or high risk, according to the standard interpretation of the instrument (31, 32).

Psychological resilience was measured using the English version of the Brief Resilient Coping Scale (BRCS), a validated four-item self-report instrument rated on a 5-point Likert scale, with a total score ranging from 4 to 20. According to established scoring guidelines, resilience levels were categorized as low (4–13), moderate (14–16), or high (17–20) (33).

### 2.3 Ethical considerations

Ethical approval to conduct this study was granted by the Institutional Review Board (IRB) of the College of Medicine, King Saud University (Research Project No. E-25-9740). The collected data were anonymous and confidential, with no names, student IDs, or any other identifying information, consistent with the IRB-approved protocol. All the participants were provided with detailed information outlining the study objectives and the voluntary nature of their participation. Formal informed consent was obtained electronically from all the participants by clicking the “agree to participate” button after reading the informed consent.

In the consent section of the electronic survey, we clearly displayed the principal investigator’s (PI) name and contact information, noting that the PI is a psychiatrist. In the consent form, we emphasized and encouraged participants who were experiencing any distress to contact the PI, visit the emergency department, or reach out to a psychiatric clinic. Clinical assessment and access to mental health services were provided to all the participants who sought assistance. Furthermore, participants who expressed feelings of distress during phone calls, which were meant to explain and invite them to participate in the research, were also connected with mental health services.

### 2.4 Statistical data analysis

Statistical analyses were performed using SPSS, version 28 (IBM Corp., Armonk, NY, USA). Continuous variables are presented as means and standard deviations (SDs). Differences in continuous variables were analyzed using an independent sample t-test. Categorical variables are presented as frequencies and percentages. Associations between categorical variables were analyzed using the chi-square test or exact test, as appropriate. Multivariate logistic regression analysis was performed to identify factors associated with moderate-to-high suicidality risk. Candidate predictors were first tested using univariate logistic regression; variables with p < 0.20 were considered for inclusion in the multivariable model. Model fit was evaluated using the Hosmer–Lemeshow goodness-of-fit test and Nagelkerke pseudo-R^2^. A two-tailed p-value < 0.05 was considered statistically significant.

## 3 Results

### 3.1 Participant characteristics

In total, 528 students were contacted. Of these, 508 agreed to participate, and 450 provided complete responses (*N* = 450; 199 males [44.2%, 95% CI: 39.6–48.8%] and 251 females [55.8%, 95% CI: 51.2–60.4%]). The distribution of universities was as follows: KSU (n=183), IMSIU (n=110), vision colleges (n=80), and DAU (n=77). Participants were predominantly Saudi nationals (91.6%, 95% CI: 89.0–94.1%) and single (96.9%). The majority of patients were aged 21–22 years (44.0%). Most students lived with their families (83.8%), and 76.3% reported satisfaction with their household income. Most of the participants attended government universities (65.1%) and were in the clinical training phase (58.9%). Academic failure was reported by 21.6% (95% CI: 18.0–25.6%). Chronic medical illness was uncommon (5.3%, 95% CI: 3.6–7.8%). Adequate physical activity was maintained by 53.6%, and adequate sleep (≥7 hours) was reported by 56.9% (95% CI: 52.3–61.4%). Participant characteristic details are presented in **Table 1** (see also **Supplementary Tables 1, 2** for expanded sociodemographic and academic details).

**Table 1:** Summary of participant characteristics (*N* = 450).

Variable	Category	n	%	95% CI
Age	≤20 years	67	14.9%	11.9–18.5%
	21–22 years	198	44.0%	39.5–48.6%
	23–24 years	116	25.8%	22.0–30.0%
	≥25 years	69	15.3%	12.3–18.9%
Gender	Male	199	44.2%	39.6–48.8%
	Female	251	55.8%	51.2–60.4%
Nationality	Saudi	412	91.6%	88.6–93.8%
	Non-Saudi	38	8.4%	6.2–11.4%
Marital status	Single	436	96.9%	94.8–98.1%
	Married/Engaged	14	3.1%	1.8–4.9%
Living arrangement	With family	377	83.8%	80.1–87.0%
	With friends	28	6.2%	4.2–9.0%
	Alone	45	10.0%	7.4–13.3%
Income satisfaction	Satisfied	343	76.3%	72.1–80.0%
	Neutral	53	11.8%	9.0–15.2%
	Dissatisfied	54	12.0%	9.2–15.5%
University type	Governmental	293	65.1%	60.7–69.3%
	Private	157	34.9%	30.7–39.3%
Training phase	Preclinical	185	41.1%	36.7–45.7%
	Clinical	265	58.9%	54.3–63.3%
Ever failed a course	Yes	97	21.6%	18.0–25.6%
Chronic illness	Yes	24	5.3%	3.6–7.8%
Physical activity	<50 min/week	209	46.4%	41.9–51.1%
	≥50 min/week	241	53.6%	48.9–58.1%
Sleep adequacy	Adequate (≥7 h)	256	56.9%	52.3–61.4%
	Inadequate (<7 h)	194	43.1%	38.6–47.7%
Nicotine use	Yes	10	2.2%	1.2–4.1%
Illegal substance use	Yes	18	4.0%	2.5–6.2%

CI, confidence interval. 95% CIs are Wilson score intervals.

### 3.2 Mental health history, psychosocial factors, and suicidal behaviors

A previous diagnosis of a mental illness was reported by 19.1% (95% CI: 15.7–23.0%) of participants (*n* = 86). Among these students, generalized anxiety disorder (60.5%) and depression/dysthymia (47.7%) were the most common diagnoses. Psychiatric medication use was reported by 18.2% (95% CI: 14.9–22.1%), and 18.0% (95% CI: 14.7–21.8%) had received prior psychotherapy. Only 9.1% (95% CI: 6.8–12.1%) were currently receiving treatment. Additionally, 25.1% (*n* = 113) perceived easy access to mental health services.

A major life stressor in the preceding year was reported by 83.6% (95% CI: 79.8–86.7%), predominantly academic-related. Parental neglect was reported by 26.9% (95% CI: 23.0–31.2%), and 31.8% (95% CI: 27.6–36.2%) experienced at least one form of abuse. Lifetime passive death wishes were reported by 36.0% (95% CI: 31.7–40.5%), and lifetime suicidal ideation by 15.8% (95% CI: 12.7–19.4%). Persistent thoughts of death in the past 12 months were reported by 21.1% (95% CI: 17.6–25.1%). Lifetime self-harm behaviors occurred in 9.3% (95% CI: 7.0–12.4%) of the participants. These data are presented in **Table 2**. The most common method of self-harm was self-cutting or self-mutilation (69.0%), followed by overdosing (23.8%). The most frequently reported motives were escape from reality or self-punishment (64.3%), depression or hopelessness (52.4%), and pain relief (42.9%).

**Table 2:** Mental health history, psychosocial factors, and suicidal behaviors (*N* = 450).

Variable	Response	n	%	95% CI
Mental health history				
Previous psychiatric diagnosis	Yes	86	19.1%	15.7–23.0%
History of psychiatric medication use	Yes	82	18.2%	14.9–22.1%
History of psychotherapy	Yes	81	18.0%	14.7–21.8%
Currently receiving psychiatric treatment	Yes	41	9.1%	6.8–12.1%
Access to care				
Easy access to mental health services	Yes	113	25.1%	21.3–29.3%
Life stressors & psychosocial factors				
Exposure to major life stressor (past year)	Yes	376	83.6%	79.8–86.7%
Experienced any form of abuse	Yes	143	31.8%	27.6–36.2%
Parental neglect	Yes	121	26.9%	23.0–31.2%
Satisfaction with friendships	Yes	297	66.0%	61.6–70.4%
Family history				
First-degree relative with psychiatric disorder	Yes	103	22.9%	19.2–26.9%
Family history of suicide attempt	Yes	24	5.3%	3.6–7.8%
Family history of suicide	Yes	3	0.7%	0.2–1.9%
Suicidal thoughts (lifetime)				
Passive death wishes	Yes	162	36.0%	31.7–40.5%
Suicidal ideation	Yes	71	15.8%	12.7–19.4%
Persistent thoughts of death (past 12 months)	Yes	95	21.1%	17.6–25.1%
Self-harm behaviors (lifetime)				
At least one self-harm behavior	Yes	42	9.3%	7.0–12.4%
Peer suicidality				
Friend attempted or died by suicide	Yes	44	9.8%	7.4–12.9%

CI, confidence interval. 95% CIs are Wilson score intervals.

#### 3.2.1 Depressive symptom severity

The mean PHQ-9 score was 8.31 ± 6.07. The severity distribution (*N* = 450) was: no depression, 31.1% (*n* = 140); mild, 30.2% (*n* = 136); moderate, 22.2% (*n* = 100); moderately severe, 10.7% (*n* = 48); severe, 5.8% (*n* = 26). Overall, 38.7% (95% CI: 34.3–43.2%) had moderate-to-severe depressive symptoms (PHQ-9 ≥ 10), and 16.4% (95% CI: 13.3–20.2%) met the criteria for moderately severe-to-severe depression (PHQ-9 ≥ 15). The most frequently reported symptoms were fatigue (mean: 1.42 ± 0.99), sleep disturbance (1.23 ± 1.04), and loss of interest (1.04 ± 0.92). The item-level responses are shown in **Supplementary Table 3**.

#### 3.2.2 Anxiety symptom severity

The mean GAD-7 score was 7.27 ± 5.86. Anxiety severity distribution (*N* = 450) was: no anxiety, 38.2% (*n* = 172); mild, 29.6% (*n* = 133); moderate, 18.9% (*n* = 85); and severe, 13.3% (*n* = 60). In total, 61.8% (95% CI: 57.2–66.1%) reported any anxiety symptoms, and 32.2% (95% CI: 28.1–36.7%) had moderate-to-severe anxiety. The most frequently reported symptom was excessive worrying (gad3 mean: 1.30 ± 1.08). The item-level data are presented in **Supplementary Table 3**.

#### 3.2.3 Resilience

The mean BRCS score was 14.24 ± 3.31. Resilience was distributed (*N* = 450) as: low (≤13), 38.0% (*n* = 171); medium (14–16), 38.0% (*n* = 171); high (≥17), 24.0% (*n* = 108). The item-level data are shown in **Supplementary Table 4**.

#### 3.2.4 Suicidality assessment (C-SSRS)

Suicidality was assessed over the previous month (*N* = 450). A wish to die was reported by 28.0% (95% CI: 24.1–32.3%), and suicidal ideation was reported by 8.2% (95% CI: 6.0–11.1%). Among the 37 participants with suicidal ideation, 73.0% had considered a method (95% CI: 57.0–84.6%), 56.8% reported intent without a specific plan (95% CI: 40.9–71.3%), and 18.9% reported intent with a specific plan (95% CI: 9.5–34.2%). A lifetime suicide attempt or preparatory behavior was reported by 4.9% (95% CI: 3.3–7.3%) of all participants. Overall, 90.0% were classified as no/low risk (*n* = 405), 1.8% as moderate risk (*n* = 8), and 8.2% as high risk (*n* = 37). These findings are summarized in **Table 3**.

**Table 3:** C-SSRS suicidality assessment results (*N* = 450).

C-SSRS Item	No	Yes	95% CI of Yes
Suicidal thoughts and behaviors (N = 450)			
Wish to be dead	324 (72.0%)	126 (28.0%)	24.1–32.3%
Suicidal ideation (any)	413 (91.8%)	37 (8.2%)	6.0–11.1%
Thought about method (among those with ideation; n = 37)	10 (27.0%)	27 (73.0%)	57.0–84.6%
Intent without specific plan (among those with ideation; n = 37)	16 (43.2%)	21 (56.8%)	40.9–71.3%
Intent with specific plan (among those with ideation; n = 37)	30 (81.1%)	7 (18.9%)	9.5–34.2%
Lifetime suicide attempt or preparatory behavior (N = 450)	428 (95.1%)	22 (4.9%)	3.3–7.3%
Suicidality risk classification (N = 450)			
No/Low risk	405 (90.0%)		86.9–92.4%
Moderate risk	8 (1.8%)		0.9–3.5%
High risk	37 (8.2%)		6.0–11.1%

C-SSRS, Columbia-Suicide Severity Rating Scale. All questions refer to the past month unless otherwise indicated. 95% CIs are Wilson score intervals. Suicidality risk classification follows standard C-SSRS scoring.

### 3.3 Bivariate associations with suicidality risk.

Among participants classified as moderate-to-high suicidality risk (*n* = 45, 10.0%, 95% CI: 7.6–13.1%), several factors were significantly elevated compared with the no/low-risk group (*n* = 405). Female students were more prevalent in the high-risk group (71.1% vs. 54.1%, *p* = 0.029), as was physical inactivity (66.7% vs. 44.2%, *p* = 0.004). Prior psychiatric diagnoses (42.2% vs. 16.5%, *p* < 0.001), psychiatric medication use (37.8% vs. 16.0%, *p* < 0.001), prior psychotherapy (33.3% vs. 16.3%, *p* = 0.005), and academic failure (37.8% vs. 19.8%, *p* = 0.005) were more prevalent in the high-risk group. Regarding psychosocial factors, parental neglect (57.8% vs. 23.5%, *p* < 0.001), any form of abuse (71.1% vs. 27.4%, *p* < 0.001), family history of psychiatric disorders (42.2% vs. 20.7%, *p* = 0.001), and family history of suicide attempts (15.6% vs. 4.2%, *p* = 0.001) were all significantly more common. Friendship satisfaction was significantly lower in the higher-risk group (mean 3.11 ± 1.32 vs. 3.93 ± 0.97, *p* < 0.001). Depression, anxiety, and resilience according to suicidality risk are presented in **Table 4**, and the key bivariate associations for the categorical variables are provided in **Supplementary Table 5**.

**Table 4:** Depression, anxiety, and resilience by suicidality risk (*N* = 450).

Item	No/low risk (n = 405)	Moderate/high risk (n = 45)	p-value
Depression (PHQ-9)			
PHQ-9 score (mean ± SD)	7.64 ± 5.49	14.40 ± 7.57	< 0.001
No depression (0–4)	136 (33.6%)	4 (8.9%)	< 0.001
Mild depression (5–9)	126 (31.1%)	10 (22.2%)	
Moderate depression (10–14)	90 (22.2%)	10 (22.2%)	
Moderately severe depression (15–19)	40 (9.9%)	8 (17.8%)	
Severe depression (20–27)	13 (3.2%)	13 (28.9%)	
Anxiety (GAD-7)			
GAD-7 score (mean ± SD)	6.85 ± 5.71	11.02 ± 5.99	< 0.001
No anxiety (0–4)	167 (41.2%)	5 (11.1%)	0.001
Mild anxiety (5–9)	116 (28.6%)	17 (37.8%)	
Moderate anxiety (10–14)	73 (18.0%)	12 (26.7%)	
Severe anxiety (15–21)	49 (12.1%)	11 (24.4%)	
Psychological resilience (BRCS)			
BRCS score (mean ± SD)	14.31 ± 3.30	13.60 ± 3.33	0.170
Low resilience (≤13)	150 (37.0%)	21 (46.7%)	0.447
Medium resilience (14–16)	156 (38.5%)	15 (33.3%)	
High resilience (≥17)	99 (24.4%)	9 (20.0%)	

PHQ-9, Patient Health Questionnaire-9; GAD-7, Generalized Anxiety Disorder-7; BRCS, Brief Resilient Coping Scale. Continuous data presented as mean ± SD; categorical data as frequency (%). p-values from independent-samples t-test (continuous) or chi-square test (categorical).

### 3.4 Multivariable logistic regression: independent predictors of suicidality risk

Three factors were associated with moderate-to-high suicidality risk in the multivariable model (**Table 5**; Nagelkerke *R*^2^ = 0.274; Hosmer–Lemeshow χ^2^(8) = 3.66, *p* = 0.887; AUC = 0.820). Severe depression (PHQ-9:20–27) was the strongest predictor of suicidality (aOR = 14.54, 95% CI: 3.09–68.55, *p* = 0.001). Lesser depression severity categories did not reach significance: mild (aOR = 1.59, 95% CI: 0.45–5.60, *p* = 0.472), moderate (aOR = 1.97, 95% CI: 0.52–7.47, *p* = 0.318), and moderately severe (aOR = 2.88, 95% CI: 0.65–12.80, *p* = 0.166). Any history of abuse (sexual, physical, or emotional) was associated with higher odds of suicidality (aOR = 4.22, 95% CI: 2.02–8.84, *p* < 0.001). Mild anxiety (GAD-7:5–9) was associated with suicidality (aOR = 3.29, 95% CI: 1.08–10.01, *p* = 0.036), whereas moderate-to-severe anxiety (GAD-7:10–21) was not significant after adjustment (aOR = 1.58, 95% CI: 0.45–5.51, *p* = 0.472). This pattern may reflect a statistical association between severe anxiety and depression; participants with moderate-to-severe anxiety were disproportionately represented in the severe depression category, and depression may have captured the shared variance with anxiety in the multivariable model. Being female was significant in the univariate analysis (OR = 2.09, 95% CI: 1.07–4.10, *p* = 0.032), but the effect was attenuated to non-significance after adjustment (aOR = 1.57, 95% CI: 0.74–3.35, *p* = 0.239).

**Table 5:** Logistic regression analysis: predictors of moderate-to-high suicidality risk (*N* = 450).

Variable	Univariate analysis			Multivariable analysis		
	OR	95% CI	p-value	aOR	95% CI	p-value
Age						
≤20 years (Ref.)	1.00	—	—			
21–22 years	2.49	0.84–7.39	0.101			
23–24 years	1.49	0.45–4.94	0.518			
≥25 years	0.97	0.23–4.04	0.966			
Gender						
Male (Ref.)	1.00	—	—	1.00	—	—
Female	2.09	1.07–4.10	0.032	1.57	0.74–3.35	0.239
Physical activity						
<50 min/week (Ref.)	1.00	—	—	—	—	—
50–100 min/week	0.30	0.12–0.74	0.009	—	—	—
101–149 min/week	0.13	0.02–1.00	0.050	—	—	—
≥150 min/week	0.78	0.34–1.80	0.564	—	—	—
Mental health & psychosocial factors						
Psychiatric diagnosis	3.69	1.93–7.04	< 0.001	—	—	—
History of psychiatric medications	3.18	1.64–6.14	0.001	—	—	—
Academic failure (≥1 course)	2.47	1.29–4.73	0.007	—	—	—
Family history of psychiatric disorder	2.79	1.47–5.29	0.002	—	—	—
Family history of suicide attempt	4.20	1.64–10.78	0.003	—	—	—
Parental neglect	4.47	2.37–8.42	< 0.001	—	—	—
Abuse (any form)	6.52	3.30–12.88	< 0.001	4.22	2.02–8.84	< 0.001
Friendship satisfaction (per unit)	0.51	0.38–0.67	< 0.001	—	—	—
Depression severity (PHQ-9)						
No depression, 0–4 (Ref.)	1.00	—	—	1.00	—	—
Mild, 5–9	2.70	0.83–8.82	0.101	1.59	0.45–5.60	0.472
Moderate, 10–14	3.78	1.15–12.41	0.029	1.97	0.52–7.47	0.318
Moderately severe, 15–19	6.80	1.95–23.76	0.003	2.88	0.65–12.80	0.166
Severe, 20–27	34.00	9.67–119.48	< 0.001	14.54	3.09–68.55	0.001
Anxiety severity (GAD-7)						
No anxiety, 0–4 (Ref.)	1.00	—	—	1.00	—	—
Mild anxiety, 5–9	4.89	1.76–13.64	0.002	3.29	1.08–10.01	0.036
Moderate–severe anxiety, 10–21	6.30	2.33–17.03	< 0.001	1.58	0.45–5.51	0.472

OR, odds ratio; aOR, adjusted odds ratio; CI, confidence interval; Ref., reference category. Bold aOR values indicate independent predictors significant at p < 0.05 in the multivariable model. Model diagnostics: Nagelkerke R^2^ = 0.274; Hosmer–Lemeshow χ^2^(8) = 3.66, p = 0.887.

## 4 Discussion

Suicide among medical students is an issue of growing global concern. This study examined the prevalence of suicidal behavior and its association with depression, anxiety, and other risk factors among medical students in Saudi Arabia (34). A substantial proportion of the participants reported lifetime suicidality, with 36% reporting lifetime death wishes and 15.8% reporting lifetime suicidal ideation. In contrast, the C-SSRS indicated notable recent suicidality during the past month, with 28% reporting death wishes and 8.2% reporting suicidal ideation. These rates are markedly higher than those observed in the general population, where suicidal ideation, for instance, is estimated at 4.9% (4). Furthermore, when compared to physicians, who reported lower rates of 12-month suicidal ideation (9.7%) (35), our findings suggest that medical students may represent a particularly high-risk group. This elevated vulnerability may be attributed to academic pressure, career uncertainty, and developing coping mechanisms during training. Internationally, our findings are broadly consistent with those of studies conducted on medical students in China and Italy (36, 37), reinforcing the global nature of the issue. Similarly, recent evidence from Tunisia reported substantial rates of suicidal ideation and suicide attempts among medical students, further highlighting the persistent burden of suicidality across diverse educational and cultural settings (38).

Furthermore, a cross-national study comparing suicide reporting patterns across Muslim-majority and non-Muslim-majority countries suggested that the relatively low reported suicide rates in many Muslim-majority societies may not fully reflect the true burden of suicidality, partly due to social stigma, religious considerations, and concerns regarding family reputation (39). Consequently, despite the substantial prevalence observed in our sample, the actual burden of suicidality may still be underestimated. As such, the interpretation of these findings within the Saudi context must take into account cultural, religious, and social influences (40). For instance, recent evidence from Saudi Arabia suggests that the mental illness stigma remains an important barrier to disclosure and help-seeking behaviors, particularly among young adults (40). Another Saudi study found that a stigmatizing attitude toward mental illness remained common among medical students. Moreover, this was associated with a less favorable attitude toward seeking professional psychological support (41). Furthermore, another Saudi study indicated that stigma toward mental illness remained prevalent among Saudi medical students even after completing the psychiatric course curriculum during medical school, suggesting that increased psychiatric knowledge alone may be insufficient to substantially overcome a negative attitude toward mental illness and help-seeking behaviors (42). Within this context, concerns relating to professional image, perceived weakness, and the potential social consequences of disclosure may contribute to the underreporting of suicidal thoughts and psychological distress. Ultimately, this results in an underestimation of the true burden of suicidality among medical students (41, 42).

Our findings further indicate a high prevalence of depressive and anxiety symptoms among the participants, with 38.7% exhibiting moderate-to-severe depressive symptoms and 32.2% exhibiting moderate-to-severe anxiety. These results closely align with large-scale international meta-analyses that included over 122, 000 medical students from 43 countries and reported an overall depression prevalence of 27.2% (22). Similarly, another meta-analysis reported a global pooled prevalence of 33.8% for anxiety, underscoring the universality of emotional distress among medical students (43). A recent systematic review demonstrated that depression, anxiety, and suicidal ideation remain highly prevalent among medical students worldwide despite increasing awareness and mental health initiatives (44). The slightly higher prevalence observed in our study may reflect cultural differences or expectations, and perhaps limited access to mental health services. Beyond academic demands, medical students in Saudi Arabia are exposed to a highly demanding educational environment characterized by intensive coursework, frequent examinations, and concerns regarding future professional performance, all of which have been identified as important contributors to psychological distress among medical students (41). In another Saudi study at Jazan University, 38.2% of students exhibited depressive symptoms, and 56.4% demonstrated moderate to extremely severe anxiety, indicating alarmingly high emotional distress (19). Similarly, studies from Umm Al-Qura University and Dammam consistently reported elevated rates of depression and anxiety among medical students, exceeding those of their non-medical peers (18, 45). Notably, due to the cross-sectional design of our study, the observed associations between depression, anxiety, and suicidality should not be interpreted as causal. Our findings reflect associations, rather than directional or causal pathways.

An important finding of this study was the central role of depression and anxiety as independent predictors of suicidality. Students with severe depressive symptoms demonstrated more than twelvefold increased odds of suicidal ideation. This observation is strongly supported by the international literature. For instance, a meta-analysis reported that depression increased the odds of suicidal ideation by nearly sevenfold and suicide attempts by more than ninefold among medical students (34). Similarly, a meta-analysis conducted by Kaggwa et al. (20) concluded that depression was consistently associated with suicide attempts (20). In Saudi Arabia, Madadin et al. (46) found that depressive symptoms and academic dissatisfaction independently predict suicidal ideation among medical students (46). These regional findings strongly support our results and reinforce the central mediating role of depression.

In our study, a mild level of anxiety was associated with an increase in suicidality risk. Previous research also indicated that anxiety may potentiate suicidal ideation through sustained psychological distress, emotional exhaustion, and impaired coping mechanisms (22, 47). Among medical students in this study, moderate and severe levels of anxiety were not found to be statistically significantly associated with suicide risk. We propose two explanations for our finding, referring to the mild level. First, individuals with more pronounced anxiety symptoms may be more likely to recognize their difficulties and seek professional support, potentially reducing progression to suicidal behaviour, as shown in some studies (48, 49). Second, our findings might indicate that other unmeasured factors, such as personality-related characteristics, may indirectly increase suicidality risk in those with mild anxiety (50).

Furthermore, the coexistence of depression and anxiety is likely to exert a synergistic effect that amplifies vulnerability to suicide. The high prevalence of depression and anxiety among medical students has been widely attributed to academic overload, competitive learning environments, frequent examinations, sleep deprivation, and the fear of academic failure. One study demonstrated that medical students exhibit significantly higher levels of psychological distress than their age-matched peers in the general population, suggesting that medical training itself is a significant psychological stressor (47). Our findings reinforce this conceptualization. This pattern is further supported by Saudi studies showing significantly higher rates of depression and psychological distress among medical students than among their non-medical peers, both at Umm Al-Qura University and in Dammam, highlighting the substantial mental health burden associated with medical education (18, 45). Overall, the overwhelming consistency of the evidence underscores that targeting depressive and anxiety symptoms is critical for suicide prevention among medical students.

In our study sample, being female was significantly associated with increased suicidal ideation and behavior. Although males generally demonstrate higher rates of completed suicide, females tend to exhibit a higher prevalence of suicidal ideation and non-fatal attempts (51), potentially reflecting heightened stress sensitivity and sociocultural pressures. These findings have been consistently reported in several studies. In Ethiopia, female students demonstrated five-fold higher odds of suicidal ideation and eight-fold higher odds of suicide attempts (9). Similarly, a systematic review and meta-analysis identified being female as a significant predictor of suicidal ideation across pooled data sets (52). Comparable findings have been observed in China and Pakistan, where females consistently reported a higher prevalence of suicidal thoughts and attempts (13, 14). Existing Saudi Arabian studies further support this trend. This reflects the consistency of gender-based vulnerability in the Saudi context (19, 45). We hypothesized that the observed sex disparity is likely driven by a combination of biological, psychological, and sociocultural factors. Interventions targeting female students’ mental health, including stress management, counseling, and supportive academic environments, may help to mitigate this elevated risk.

Academic stress emerged as the predominant stressor affecting 92% of the students in this study. Bivariate analysis revealed that failing a course significantly increased suicide risk. However, this association disappeared after adjusting for depression and anxiety. This suggests that the relationship between suicidality and academic failure may be explained by mental health rather than as an independent risk factor. These findings align with the literature linking academic stressors to suicidality through psychological distress. Okechukwu et al. reported that academic stress is associated with suicidal ideation only at low to moderate coping levels, indicating that coping capacity mediates this relationship (53). We contend that the stress-coping model (54) can be used to explain this further. When perceived demands exceed available resources, students develop depression and anxiety, which in turn, elevates suicide risk (55). A case-control study has validated this framework. In that study, depression and anxiety fully mediated the association between stressful life events and poor academic adjustment (56). These findings underscore the urgent need for educational reforms that prioritize academic support systems and learning environments that balance achievement with well-being.

One of the clinically significant findings of our study was the strong association among childhood abuse, parental neglect, and suicidality. Students who reported a history of abuse exhibited a more than three-fold increase in suicide risk, underpinning the enduring psychological impact of early life adversity. This observation aligns closely with evidence from Saudi Arabia and internationally. In Dammam, parental neglect and physical abuse were identified as major independent predictors of suicidal ideation (46). Similarly, at Umm Al-Qura University, parental neglect emerged as one of the strongest predictors of suicidality (26). These findings suggest that family environment plays a critical role in shaping emotional vulnerability. International literature further reinforces this association. A large-scale Ethiopian study found that adverse childhood experiences and poor family support were consistently associated with increased suicidal ideation among university students (9). Collectively, these findings emphasize that abuse and parental neglect have a far-reaching impact but are frequently overlooked as determinants of suicidality. Early identification, trauma-informed counseling, and strengthening family support may help reduce suicidality among students with a history of childhood adversity.

Dissatisfaction with friendships has emerged as a significant risk factor for suicide. However, following multivariate analysis, the relationship with suicide was not significant, suggesting that the primary effect of social support occurs through symptom modulation. These results are consistent with those of Alhassan et al. (35) in the Middle East, North Africa, and Turkey (MENAT) region and Puthran et al. (57), both documenting that social connections, family bonds, and peer support were inversely associated with suicidality (35, 57). In contrast, among Korean medical students, dissatisfaction with friendships remained a risk factor even after adjusting for depression and anxiety. These divergent results can be explained by differences in methodological approaches (58).

Furthermore, our results demonstrated a significant association between physical inactivity and suicidality, whereby students reporting minimal exercise showed a high prevalence of suicidality. These findings are in line with evidence documenting inverse relationships between physical activity frequency and suicide risk factors, highlighting the current vulnerability of medical students (59). Medical students encounter unique time constraints that prevent them from maintaining healthy behaviors and rigorous academic demands, tight study schedules, intensive clinical rotations, and frequent exams, leaving limited time for physical activity (60). The meta-analysis by Rotenstein et al. (22) documented that the mean absolute increase in depressive symptoms following the onset of medical school education was 13.5%, coinciding with lifestyle deterioration, including reduced exercise and social engagement (22). Based on these findings, we recommend initiatives within medical educational institutes that address time-related barriers to physical activity to safeguard students’ physical and mental well-being. For example, scheduling protected wellness periods, providing on-campus fitness facilities, and incorporating exercise into formal curricula.

Finally, resilience in our study, measured by the BRCS, indicated that 38% of students had low resilience, 38% had medium resilience, and 24% had high resilience. These results show that resilience does not demonstrate a statistically significant protective effect against suicidality, contradicting the abundant local and global literature (25, 58, 61–63). This inconsistency warrants careful interpretation and several explanations may account for our findings. First, the severity of depression and anxiety in our sample may have exceeded the protective elements of resilience. Second, the concept of resilience may have manifested differently within the cultural context of the sample. Third, our study may have lacked the statistical power to detect the protective effect of resilience, given the relatively small number of students with moderate to high suicide risk. Additionally, a recent systematic review and meta-analysis of 21 studies involving medical students and resilience training interventions revealed small-to-moderate effects on resilience and moderate effects on stress management (64).

### 4.1 Strengths and limitations

This study has several strengths that enhance the reliability and generalizability of its findings. First, the assessment of mental health outcomes relied on highly validated and globally recognized scales (i.e., the PHQ-9, GAD-7, C-SSRS, and BRCS), facilitating direct comparability with international literature. Second, this study employed a multicenter design and recruited participants from a diverse range of academic institutions in Riyadh, including both public (government) and private universities. Unlike single-center studies, which may reflect the specific environment of one institution, this broad sampling strategy mitigates selection bias and improves the external validity of the results, thereby offering a more accurate representation of the medical student population. Additionally, this study provides insights into practical recommendations and applications of well-being programs and mental health support for medical students in the KSA. Finally, the breadth of the variables allowed the execution of a valid multivariate regression analysis, permitting adjustment for potential confounders. This analytical rigour is critical for isolating independent predictors of suicidality, distinguishing them from factors that are merely correlated, and thereby identifying precise targets for future preventive interventions.

This study also has some limitations. The cross-sectional design measured exposure and outcomes simultaneously, preventing temporal ordering and limiting causal interpretation. Moreover, causal relationships and bidirectionality between depression, anxiety, and suicidality cannot be inferred. The observed associations represent correlations only in this study. Therefore, future research should employ longitudinal or prospective designs to determine temporal ordering and clarify potential causal mechanisms. Another limitation is the reliance on self-reported data, which may affect data precision. Furthermore, self-reported data might be affected by social desirability bias, in which participants might modify their responses to appear more acceptable, further introducing the possibility of data inaccuracy. We recommend that future Saudi studies consider collecting data using objective measures, such as clinical assessments, or independently verifiable sources, including clinical records and behavioral or observable indicators. Moreover, questions about past events are vulnerable to recall bias, reducing the precision of the reported information, and shorter recall periods or validated historical records are recommended for future studies. Although this was a multicenter study, all participants were from a single geographical region, the city of Riyadh. This geographical sampling bias may limit the generalizability of our findings to medical students across other areas of Saudi Arabia. We recommend that future Saudi studies include a broader and more diverse geographic area to achieve better representation and improve external validity. Additionally, the strong correlation between depression and anxiety scores may cause multicollinearity in multivariate models, reducing the precision of individual estimates. Future analyses may use alternative modeling strategies or factor-based approaches. Nevertheless, residual confounding from unmeasured factors, such as personality traits, may influence the observed associations. Therefore, future studies should incorporate a broader range of potential confounders to enhance analytical robustness.

## 5 Conclusion

This study aimed to measure the prevalence and risk factors of suicidality among medical students in Riyadh, Saudi Arabia. The findings demonstrated that depression, childhood abuse, and being female emerged as powerful independent predictors of suicidality, highlighting the substantial burden of these factors among medical students in Saudi Arabia.

To the best of our knowledge, no study in Saudi Arabia has examined the effectiveness of early screening, identification, and implementation of specific interventions among medical students at risk of suicide. International evidence consistently shows that early psychological screening combined with structured mental health interventions lead to better long-term outcomes (65, 66). Furthermore, global studies indicate that medical students presenting with mild depressive or anxiety symptoms identified through early screening benefit significantly from prompt referral to mental health services (65, 67).

Within the Saudi cultural and institutional context, efforts should be made to further support student needs. Such efforts could include the development and implementation of structured academic advice, peer support programs, formal mentorship, and psychological counselling. Efforts should also entail the early detection of high-risk individuals and connecting them to mental health services for further assessment and management. When considering the most appropriate mental health services to refer students to, the factors that were found to be significant in this vulnerable group, namely, depression severity, being female, and having a history of childhood abuse, should be considered. Based on our results, we suggest that those who are found to have severe depression (using a validated screening tool for depression such as the PHQ-9) should be linked to professional mental health services to receive proper care and interventions. Regarding gender, as we found that being female was a significant predictor of suicidality, we recommend lowering the expected threshold to link to mental health services compared to males (i.e., considering linking female students to mental health services even if the depression severity is less than severe, for instance). Regarding childhood abuse, the presence of such a history warrants early consideration of mental health service linkages and interventions. Intervention-wise, specific psychological strategies to consider include psychoeducation, resilience-building programs, and trauma-informed interventions, tailored to the individual’s needs.

Ultimately, such measures could help mitigate suicide risk and foster long-term psychological well-being in this vulnerable population. Notably, while some institutions in Saudi Arabia may have begun adopting such approaches, these initiatives remain largely undocumented in the scientific literature, highlighting the need for locally generated evidence to assess their feasibility, acceptability, and long-term outcomes.

## Data Availability

The raw data supporting the conclusions of this article will be made available by the authors, without undue reservation.
