# Supplementary material for: Prevalence and risk factors of suicide and their correlation with depression and anxiety among medical students: a Saudi multicenter study
Source: Front Psychiatry. 2026 Aug 7;17:1879787. doi: 10.3389/fpsyt.2026.1879787 (PMC13493557; doi:10.3389/fpsyt.2026.1879787)
Supplement: Supplementary file 1 [file SupplementaryFile1.docx]

Supplementary Material

# Supplementary Tables

*The following supplementary tables provide expanded descriptive data supporting the main results section.*

**Supplementary Table S1. Detailed Sociodemographic Characteristics (*N* = 450)**

| **Variable** | **Category** | **n** | **%** | **95% CI** |
| --- | --- | --- | --- | --- |
| Age | ≤20 years | 67 | 14.9% | 11.9–18.5% |
|  | 21–22 years | 198 | 44.0% | 39.5–48.6% |
|  | 23–24 years | 116 | 25.8% | 22.0–30.0% |
|  | 25–26 years | 44 | 9.8% | 7.3–13.0% |
|  | 27–28 years | 16 | 3.6% | 2.2–5.7% |
|  | ≥29 years | 9 | 2.0% | 1.0–3.8% |
| Nationality | Saudi | 412 | 91.6% | 89.0–94.1% |
|  | Non-Saudi | 38 | 8.4% | 5.9–11.0% |
| Marital status | Single | 436 | 96.9% | 95.1–98.2% |
|  | Married | 14 | 3.1% | 1.8–4.9% |
| Living arrangement | With family/relative | 377 | 83.8% | 80.1–87.0% |
|  | With roommate/friend (off campus) | 14 | 3.1% | 1.8–5.2% |
|  | With roommate/friend (student housing) | 14 | 3.1% | 1.8–5.2% |
|  | Alone (student housing) | 32 | 7.1% | 5.0–10.0% |
|  | Alone (off campus) | 13 | 2.9% | 1.7–4.9% |
| Income satisfaction | Satisfied | 343 | 76.3% | 72.1–80.0% |
|  | Neutral | 53 | 11.8% | 9.0–15.2% |
|  | Dissatisfied | 54 | 12.0% | 9.2–15.5% |
| *CI = confidence interval. 95% CIs are Wilson score intervals for proportions.* | | | | |

**Supplementary Table S2. Full Academic Characteristics (*N* = 450)**

| **Variable** | **Category** | **n** | **%** | **95% CI** |
| --- | --- | --- | --- | --- |
| University type | Governmental | 293 | 65.1% | 60.7–69.3% |
|  | Private | 157 | 34.9% | 30.7–39.3% |
| Academic year | First year | 12 | 2.7% | 1.5–4.6% |
|  | Second year | 69 | 15.3% | 12.2–19.1% |
|  | Third year | 104 | 23.1% | 19.4–27.3% |
|  | Fourth year | 127 | 28.2% | 24.1–32.7% |
|  | Fifth year | 78 | 17.3% | 14.0–21.3% |
|  | Sixth year | 60 | 13.3% | 10.4–17.0% |
| Training phase | Preclinical | 185 | 41.1% | 36.7–45.7% |
|  | Clinical | 265 | 58.9% | 54.3–63.3% |
| Ever dropped a course/semester | Yes | 60 | 13.3% | 10.4–17.0% |
|  | No | 390 | 86.7% | 83.0–89.6% |
| Ever failed a course | Yes | 97 | 21.6% | 18.0–25.6% |
|  | No | 353 | 78.4% | 74.4–82.0% |
| Clear academic plans for the future | Yes | 355 | 78.9% | 74.9–82.4% |
|  | No | 95 | 21.1% | 17.6–25.1% |
| *CI = confidence interval. 95% CIs are Wilson score intervals for proportions.* | | | | |

**Supplementary Table S3. Item-Level Responses on the PHQ-9 and GAD-7 (*N* = 450)**

| **Item** | **Not at all** | **Several days** | **More than half the days** | **Nearly every day** | **Mean ± SD** |
| --- | --- | --- | --- | --- | --- |
| **PHQ-9 Items (N = 450)** | | | | | |
| Little interest or pleasure in doing things | 140 (31.1%) | 194 (43.1%) | 74 (16.4%) | 42 (9.3%) | 1.04 ± 0.92 |
| Feeling down, depressed, or hopeless | 157 (34.9%) | 201 (44.7%) | 59 (13.1%) | 33 (7.3%) | 0.93 ± 0.88 |
| Trouble falling or staying asleep, or sleeping too much | 134 (29.8%) | 149 (33.1%) | 96 (21.3%) | 71 (15.8%) | 1.23 ± 1.04 |
| Feeling tired or having little energy | 77 (17.1%) | 197 (43.8%) | 88 (19.6%) | 88 (19.6%) | 1.42 ± 0.99 |
| Poor appetite or overeating | 192 (42.7%) | 129 (28.7%) | 75 (16.7%) | 54 (12.0%) | 0.98 ± 1.04 |
| Feeling bad about yourself or that you are a failure | 179 (39.8%) | 144 (32.0%) | 69 (15.3%) | 58 (12.9%) | 1.01 ± 1.03 |
| Trouble concentrating on things | 210 (46.7%) | 132 (29.3%) | 52 (11.6%) | 56 (12.4%) | 0.90 ± 1.04 |
| Moving or speaking too slowly or being fidgety/restless | 303 (67.3%) | 88 (19.6%) | 39 (8.7%) | 20 (4.4%) | 0.50 ± 0.83 |
| Thoughts of being better off dead or of hurting yourself | 358 (79.6%) | 61 (13.6%) | 17 (3.8%) | 14 (3.1%) | 0.30 ± 0.69 |
| **Total PHQ-9 score** | | | | | **8.31 ± 6.07** |
| **GAD-7 Items (N = 450)** | | | | | |
| Feeling nervous, anxious, or on edge | 113 (25.1%) | 189 (42.0%) | 93 (20.7%) | 55 (12.2%) | 1.20 ± 0.95 |
| Not being able to stop or control worrying | 153 (34.0%) | 157 (34.9%) | 77 (17.1%) | 63 (14.0%) | 1.11 ± 1.03 |
| Worrying too much about different things | 124 (27.6%) | 157 (34.9%) | 80 (17.8%) | 89 (19.8%) | 1.30 ± 1.08 |
| Trouble relaxing | 176 (39.1%) | 145 (32.2%) | 74 (16.4%) | 55 (12.2%) | 1.02 ± 1.02 |
| Being so restless that it is hard to sit still | 252 (56.0%) | 124 (27.6%) | 42 (9.3%) | 32 (7.1%) | 0.68 ± 0.91 |
| Becoming easily annoyed or irritable | 155 (34.4%) | 166 (36.9%) | 71 (15.8%) | 58 (12.9%) | 1.07 ± 1.01 |
| Feeling afraid as if something awful might happen | 216 (48.0%) | 121 (26.9%) | 57 (12.7%) | 56 (12.4%) | 0.90 ± 1.05 |
| **Total GAD-7 score** | | | | | **7.27 ± 5.86** |
| *PHQ-9 = Patient Health Questionnaire-9; GAD-7 = Generalized Anxiety Disorder-7. Data are frequency (%) and mean ± SD.* | | | | | |

**Supplementary Table S4. Item-Level Responses on the Brief Resilient Coping Scale (BRCS; *N* = 450)**

| **Item** | Does not describe me at all | Does not describe me | Neutral | Describes me | Describes me very well | **Mean ± SD** |
| --- | --- | --- | --- | --- | --- | --- |
| I look for creative ways to alter difficult situations. | 33 (7.3%) | 48 (10.7%) | 168 (37.3%) | 133 (29.6%) | 68 (15.1%) | 3.34 ± 1.09 |
| Regardless of what happens, I believe I can control my reaction to it. | 18 (4.0%) | 51 (11.3%) | 128 (28.4%) | 154 (34.2%) | 99 (22.0%) | 3.59 ± 1.07 |
| I believe I can grow in positive ways by dealing with difficult situations. | 17 (3.8%) | 29 (6.4%) | 124 (27.6%) | 174 (38.7%) | 106 (23.6%) | 3.72 ± 1.02 |
| I actively look for ways to replace the losses I encounter in life. | 19 (4.2%) | 37 (8.2%) | 142 (31.6%) | 163 (36.2%) | 89 (19.8%) | 3.59 ± 1.03 |
| **Total BRCS score** | | | | | | **14.24 ± 3.31** |
| *BRCS = Brief Resilient Coping Scale. Items rated 1 (does not describe me at all) to 5 (describes me very well). Low resilience (≤13): 38.0%; Medium (14–16): 38.0%; High (≥17): 24.0%.* | | | | | | |

**Supplementary Table S5. Bivariate Associations Between Selected Risk Factors and Suicidality Risk**

| **Variable** | **No/Low Risk (n = 405)** | **Moderate/High Risk (n = 45)** | **p-value** |
| --- | --- | --- | --- |
| **Demographic & Lifestyle Factors** | | | |
| Female gender | 219 (54.1%) | 32 (71.1%) | **0.029** |
| Physical inactivity (<50 min/wk) | 179 (44.2%) | 30 (66.7%) | **0.004** |
| Inadequate sleep (<7 h) | 172 (42.5%) | 22 (48.9%) | 0.409 |
| **Mental Health History** | | | |
| Previous psychiatric diagnosis | 67 (16.5%) | 19 (42.2%) | **< 0.001** |
| History of psychiatric medications | 65 (16.0%) | 17 (37.8%) | **< 0.001** |
| History of psychotherapy | 66 (16.3%) | 15 (33.3%) | **0.005** |
| Academic failure (≥1 course) | 80 (19.8%) | 17 (37.8%) | **0.005** |
| **Psychosocial Factors** | | | |
| Family history of psychiatric disorder | 84 (20.7%) | 19 (42.2%) | **0.001** |
| Family history of suicide attempt | 17 (4.2%) | 7 (15.6%) | **0.001** |
| Parental neglect | 95 (23.5%) | 26 (57.8%) | **< 0.001** |
| Abuse (any form) | 111 (27.4%) | 32 (71.1%) | **< 0.001** |
| *Data are presented as frequency (%). p-values from chi-square test. Statistical significance at p < 0.05.* | | | |
